# Diagnosis and Management of Tuberculous Pleural Effusion in a Patient With Chronic Obstructive Pulmonary Disease: A Case Report

**DOI:** 10.7759/cureus.64505

**Published:** 2024-07-14

**Authors:** Abhishek G Amipara, Ankit Rangari, Babaji Ghewade

**Affiliations:** 1 Clinical Research, Datta Meghe Institute of Higher Education and Research, Wardha, IND; 2 Respiratory Medicine, Datta Meghe Institute of Higher Education and Research, Wardha, IND

**Keywords:** tuberculous empyema, chronic obstructive pulmonary disease (copd), pneumothorax, adenosine deaminase (ada), tuberculous pleural effusion (tpe)

## Abstract

A 63-year-old man had been smoking bidis for 25 years and developed tubercular empyema, further complicated by pneumothorax and other pulmonary issues. Over a period of three weeks, the individual experienced a gradual onset of symptoms, including progressive shortness of breath, cough, fever, and chest pain. Radiographic examinations revealed significant left-sided pleural effusion with consolidation and evidence of pneumothorax. Other findings included anemia, hyponatremia, substantially increased lactate dehydrogenase, and adenosine deaminase (ADA), consistent with tubercular or chronic infection. The comprehensive treatment plan involved the administration of antibiotics, antitubercular drugs, draining of the pleural fluid, nebulized bronchodilators, corticosteroids, and broad-spectrum antibiotics. The patient exhibited a positive response, showing notable clinical improvement, which was closely monitored through sequential chest X-rays and ECGs. This would continue to highlight the vital need for early tuberculosis detection in patients with chronic obstructive pulmonary disease due to clinical overlap with other diseases. To diagnose and follow up on tuberculous pleural effusion cases, it was critical to integrate both clinical and radiographic findings with laboratory data. It emphasizes the necessity for a multidisciplinary approach to improve overall treatment outcomes.

## Introduction

Tuberculous pleural effusion (TPE) is one of the most common forms of tuberculosis (TB) outside the lungs, second only to TB of the lymph nodes [1]. The low bacterial load in TPE presents challenges in diagnosis, resulting in reduced sensitivity when culturing. This intricacy is exacerbated by the overlap of symptomatology in individuals afflicted with chronic obstructive pulmonary disease (COPD), whose manifestations may mimic those of TB-associated or non-TB-associated pleural effusions, thereby heightening their vulnerability. TPE originates from the reactivation of quiescent *Mycobacterium tuberculosis* bacilli, which subsequently traverse from the pulmonary parenchyma to the pleural space [2]. This results in an immunological response characterized by the accumulation of fluid and inflammatory cells in the pleura, often leading to a type IV delayed hypersensitivity reaction. Patients with COPD and TPE should have a high index of suspicion and undergo a thorough evaluation. Pleural fluid analysis is critical for detecting adenosine deaminase (ADA), a diagnostic marker. ADA levels exceeding 40 U/L are 92% sensitive and 90% TPE-specific [3]. The clinical presentation of TPE may vary from extrapulmonary features, including relentless cough, night sweats, unexplained weight loss, and low-grade fever with an evening rise in temperature. All these features in patients with COPD can be interpreted as exacerbations of their underlying diseases. All these must be cautiously examined, especially in areas with a high prevalence of TB, by direct investigations to document whether the cause of developing pleural effusion is a TPE or another pathology. With regard to those above, the present case report aims to report a TPE incident in a patient with COPD, thus highlighting the role of a multidisciplinary approach and integration among clinical, radiological, and laboratory data for diagnosis and effective treatment.

## Case presentation

**Figure 1:**
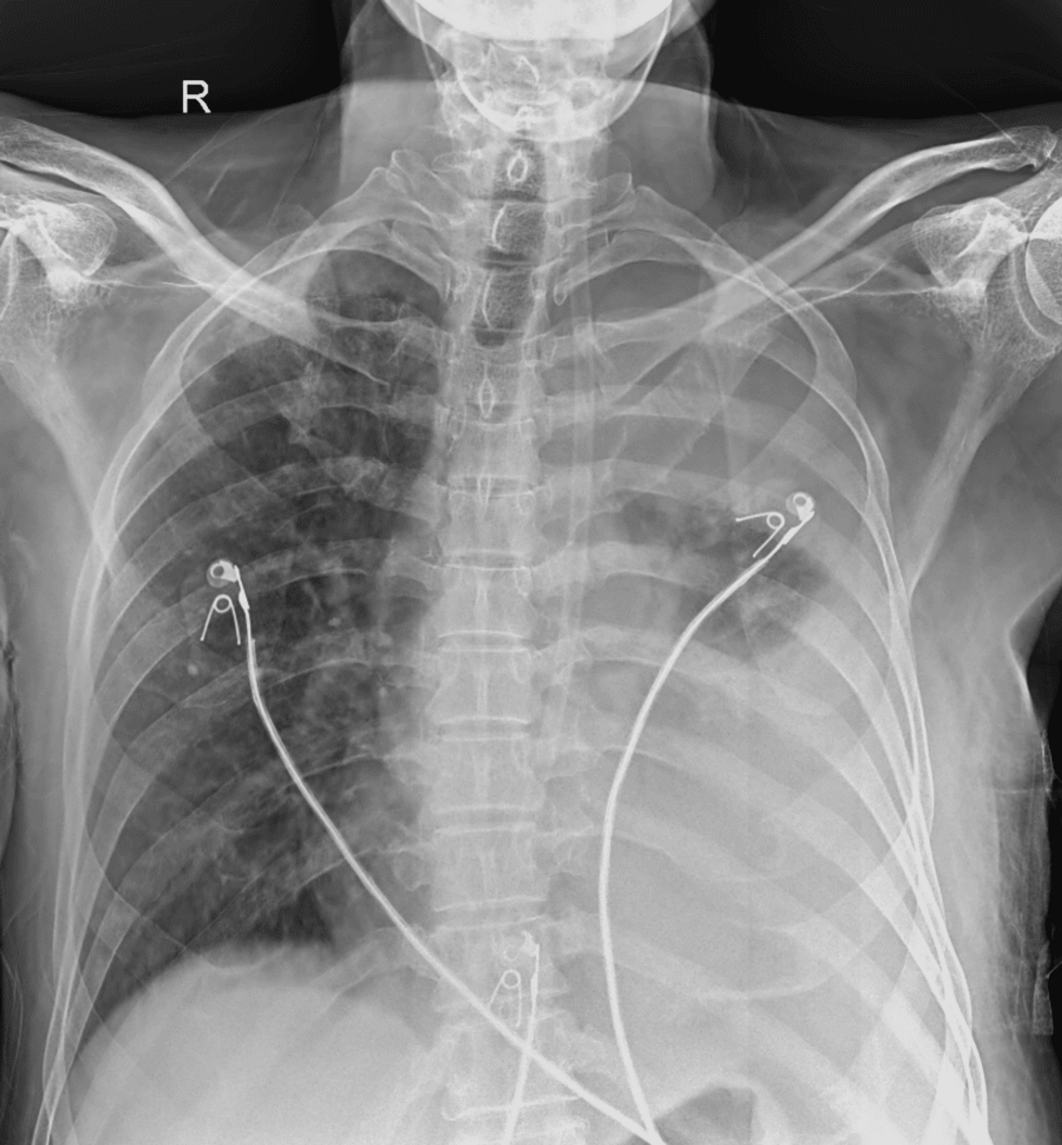
Significant opacification of the left hemithorax

A 63-year-old male farmer who has been smoking bidi for the past 30 years presented with increased breath, coughing, vomiting, fever, and chest pain that had lasted for three days. The patient was well until approximately two and a half months ago, when he began experiencing dyspnea. Initially, it was classified as grade 2, which progressively worsened to grade 3. Despite resting, the dyspnea persisted. He also reported a cough productive of mucoid sputum and constant, dull, left-sided chest pain. In the past few days, there has been an acute exacerbation of symptoms, escalating the dyspnea to grade 4, which prompted him to seek medical attention. Body temperature was 37°C as measured orally, blood pressure was 130/90 mmHg, pulse rate was 90 beats/minute, respiratory rate was 20 breaths/minute, and peripheral oxygen saturation was 94% on room air, all within normal limits. A chest examination revealed decreased air entry on the left side with stony dull note sounds on percussion. Left-sided chest expansion was asymmetrical, with diminished expansion on the same side. Also, vocal fremitus was decreased while percussion. A cardiovascular examination revealed no murmur, but normal heart sounds were heard, while a central nervous system assessment showed that the patient was conscious and oriented. The radiologic findings indicated pleural effusion in the left hemithorax, which was consistent with his presentation of breathlessness and decreased air entry over the left lower zone. A large opacification is seen in more than two-thirds of the space in the left lateral chest cavity, which was suggestive of massive pleural effusion (Figure 1).

Anemia with a hemoglobin of 9.3 g/dL was confirmed through laboratory tests, showing normal mean corpuscular volume, white blood cell count, and platelet count. The patient’s renal function was normal. Hyponatremia (sodium 132 mmol/L) and mild hypokalemia (potassium 3.1 mmol/L) were found in electrolyte analysis. Low total protein and albumin levels indicated malnutrition or chronic illnesses that he may have been suffering from for some time. The presence of high levels of lactate dehydrogenase (LDH) and ADA in both the serum and pleural fluid indicates a tubercular infection or a chronic infection. The drained pleural fluid was straw-colored and hazy, with high glucose and LDH, as well as high protein and numerous pus cells, but no organisms were seen on Gram stain smear microscopy, as shown in Tables 1, 2.

**Table 1:** Laboratory investigation LDH: lactate dehydrogenase; ADA: adenosine deaminase

Blood sample investigations	Results	Reference range
Hemoglobin (g/dL)	9.3	12-16
Mean corpuscular volume (fL)	90.2	80-96
Total white cell count (per mm^3^)	9,800	4,000-12,000
Platelet (per mm^3^)	330,000	150,000-400,000
Urea (mg/dL)	21	19-43
Sodium (mmol/L)	132	136-145
Potassium (mmol/L)	3.1	3.1-5.0
Creatinine (mg/dL)	0.9	0.66-1.25
Total protein (g/dL)	5.1	6.3-8.2
Albumin (g/dL)	2.4	3.5-5.0
LDH (mg/dL)	214	>45
ADA (U/L)	67.331	40

**Table 2:** Pleural fluid investigations HPF: high power field; TLC: total leucocyte count; DLC: differential leukocyte count

Pleural fluid investigations	Result	Reference range
Total protein (g/L)	3.6	1-2
pH	7.4	7.6-7.64
Appearance	Straw-colored	-
Glucose (mmol)	20	-
Lactate dehydrogenase (U/L)	3,297	-
Gram stain	Plenty of pus cells and no organism seen	-
Microscopy	RBCs: PLENTY/HPF; WBCs: PLENTY/HPF; TLC: approximately 2,730 cells/cumm; DLC: polymorphs (75%) and lymphocytes (25%); no organism seen	-
Culture and sensitivity	No growth after subculture from enrichment broth	-

**Figure 2:**
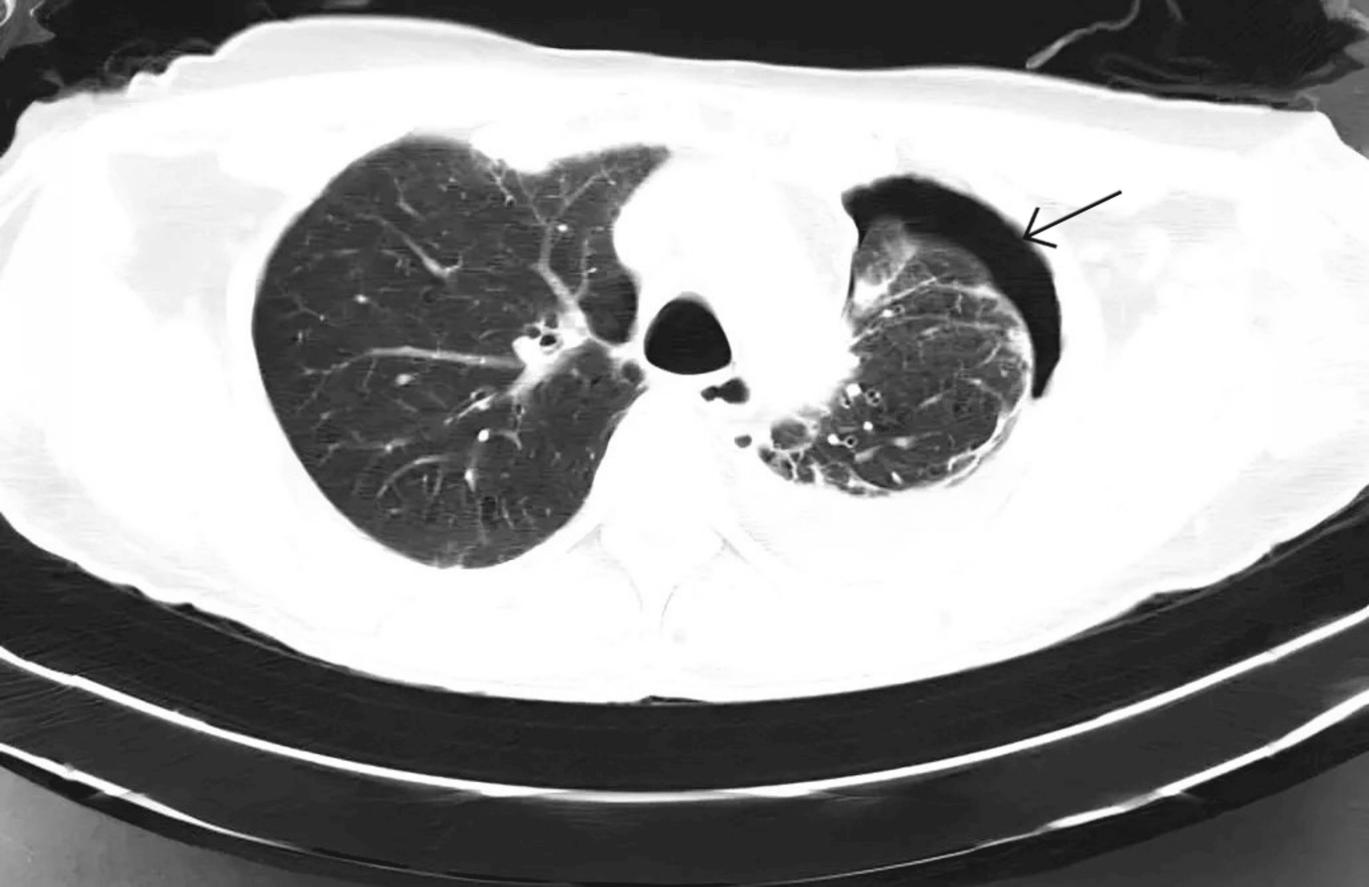
Left-sided pleural effusion, pneumothorax, and associated lung volume loss

After an X-ray and laboratory examination, a further radiological examination with high-resolution computed tomography (HRCT) was done (Figure 2). HRCT of the lungs revealed left-sided pleural effusion (approximately 160 cc) with adjacent consolidation, tractional bronchiectasis, subsegmental atelectasis, and linear fibrotic bands with lung volume loss.

The treatment included antibiotics, antitubercular therapy, and drainage of pleural fluid. A pigtail catheter removed 200 mL of pleural fluid. The patient received a 15-day course of isoniazid, rifampicin, ethambutol, pyrazinamide, nebulized bronchodilators (Duolin), and corticosteroids (Budecort). Broad-spectrum antibiotics (cefoperazone and sulbactam) were also given. The treatment was guided by targeted antimicrobial therapy, with regular chest radiography and electrocardiogram monitoring. The patient was discharged after 18 days and was advised to continue the antibiotics and additional medications to support recovery and manage residual symptoms.

## Discussion

TB is a highly disabling and lethal disease all over the globe; hence, its geography and diagnostic armamentarium should be well understood [4]. It accounts for <1% of exudative effusions in the West, but its prevalence is overwhelming in countries such as India, Pakistan, and other developing countries. In these areas, up to 80% of pleural effusions can be caused by TB [5]. The epidemiology and classical demographics of TB have been dynamic, with more information demonstrating an increased incidence of coinfection with HIV and immunosuppression [6]. In this case, the overlap of pulmonary conditions makes the diagnosis and treatment complex. The standard features of both COPD and tuberculous empyema presented by the patient were dyspnea, cough, fever, and chest pain. The diagnosis of pleural TB is often laborious, where bacilli are scarce in the pleural fluid, and the fluid is nonspecific in character. Newer diagnostic modalities are needed, such as the traditional tuberculin skin tests, Ziehl-Neelsen staining, and pleural fluid culture, which lack sensitivity in diagnosing pleural TB [7,8]. The gold standard is pleural biopsy culture. However, supplementary techniques, such as measuring ADA and interferon-gamma levels in the pleural fluid, show high sensitivity and specificity for extrapulmonary TB [9]. Laboratory results confirmed tubercular infection with elevated ADA levels in the pleural fluid and numerous pus cells without bacterial growth, consistent with the patient's history and radiographic findings. Imaging, particularly radiography and HRCT, revealed pleural effusion, pneumothorax, consolidation, and other structural changes that are crucial for understanding pulmonary involvement. In one study in which parenchymal abnormalities were found in 86% of cases, the most common CT scan findings were micronodules in the subpleural and peribronchovascular interstitium and interlobular septal thickening, suggesting lymphatic spread of TB [10]. The treatment involved antitubercular drugs, broad-spectrum antibiotics, nebulized bronchodilators, and corticosteroids. An intercostal drainage tube was effective in managing the empyema, highlighting the importance of precise antimicrobial therapy. However, this case suggests the need to consider TB in the differential diagnosis of patients with pleural effusion and pneumothorax in the face of an attendant history of smoking. However, a more truthful approach to tackling issues that address complete diagnosis includes the integration of clinical, radiological, and laboratory findings for effective management and unequivocal diagnosis. *M. tuberculosis* has not been confirmed in microbiological studies, but ample evidence has consolidated the diagnosis. This finding was significant.

## Conclusions

This case highlights the diagnostic dilemmas and management issues associated with TPE in chronic smokers with COPD. Since the symptoms in this patient had many interlacings, a very high index of suspicion and an exhaustive methodology were required to diagnose and differentiate TPE from other causes of pleural effusion. The diagnosis of TPE was thus established by the combination of clinical, radiological, and laboratory findings. In addition, the patient showed marked improvements with multidisciplinary management, involving appropriate antibiotics, antitubercular therapy, and pleural fluid drainage. This case highlights TPE as an essential differential in the diagnosis of pleural effusions in patients with COPD, even in the absence of any active pulmonary TB, and highlights the fact that a directed and holistic management policy has to be drawn up to ensure favorable outcomes.

## References

[1] McNally E, Ross C, Gleeson LE (2023). The tuberculous pleural effusion. Breathe (Sheff).

[2] Lo Cascio CM, Kaul V, Dhooria S, Agrawal A, Chaddha U (2021). Diagnosis of tuberculous pleural effusions: a review. Respir Med.

[3] Palma RM, Bielsa S, Esquerda A, Martínez-Alonso M, Porcel JM (2019). Diagnostic accuracy of pleural fluid adenosine deaminase for diagnosing tuberculosis. Meta-analysis of Spanish studies. [Article in Spanish]. Arch Bronconeumol (Engl Ed).

[4] Bañuls AL, Sanou A, Van Anh NT, Godreuil S (2015). Mycobacterium tuberculosis: ecology and evolution of a human bacterium. J Med Microbiol.

[5] Udwadia ZF, Sen T (2010). Pleural tuberculosis: an update. Curr Opin Pulm Med.

[6] Chakrabarti B, Davies PD (2006). Pleural tuberculosis. Monaldi Arch Chest Dis.

[7] Valdés L, Pose A, José ES, Vázquez JMM (2003). Tuberculous pleural effusions. Eur J Intern Med.

[8] Villegas MV, Labrada LA, Saravia NG (2000). Evaluation of polymerase chain reaction, adenosine deaminase, and interferon-gamma in pleural fluid for the differential diagnosis of pleural tuberculosis. Chest.

[9] Greco S, Girardi E, Masciangelo R, Capoccetta GB, Saltini C (2003). Adenosine deaminase and interferon gamma measurements for the diagnosis of tuberculous pleurisy: a meta-analysis. Int J Tuberc Lung Dis.

[10] Ko JM, Park HJ, Kim CH (2014). Pulmonary changes of pleural TB: up-to-date CT imaging. Chest.

